# Synthesis and crystal structure of (NH_4_)[Ni_3_(HAsO_4_)(AsO_4_)(OH)_2_]

**DOI:** 10.1107/S2056989024003487

**Published:** 2024-04-26

**Authors:** Felix Eder, Matthias Weil

**Affiliations:** a TU Wien, Institute for Chemical Technologies and Analytics, Division of Structural Chemistry, Getreidemarkt 9/E164-05-1, 1060 Vienna, Austria; University of Aberdeen, United Kingdom

**Keywords:** crystal structure, layered structure, nickel, hydrogen bonding, arsenate(V), disorder, isotypism, structural comparison

## Abstract

The title compound, ammonium trinickel(II) hydrogen arsenate arsenate di­hydroxide, was synthesized under hydro­thermal conditions. Its crystal structure is isotypic with that of K[Cu_3_(HAsO_4_)(AsO_4_)(OH)_2_] and is characterized by pseudo-hexa­gonal (001) ^2^_∞_[Ni_3_As_2_O_18/3_(OH)_6/3_O_1/1_(OH)_1/1_]^−^ layers formed from vertex- and edge-sharing [NiO_4_(OH)_2_] octa­hedra and [AsO_3.5_(OH)_0.5_] tetra­hedra as the building units. The hydrogen atom of the OH group shows occupational disorder and was refined with a site occupation factor of 1/2, indicating the equal presence of [HAsO_4_]^2–^ and [AsO_4_]^3–^ groups. Strong asymmetric hydrogen bonds between symmetry-related (O,OH) groups of the arsenate units [O⋯O = 2.588 (18) Å] as well as hydrogen bonds accepted by these (O,OH) groups from OH groups bonded to the Ni^II^ atoms [O⋯O = 2.848 (12) Å] link adjacent layers. Additional consolidation of the packing is achieved through N—H⋯O hydrogen bonds from the ammonium ion, which is sandwiched between adjacent layers [N⋯O = 2.930 (7) Å] although the H atoms could not be located in the present study. The presence of the pseudo-hexa­gonal ^2^_∞_[Ni_3_As_2_O_18/3_(OH)_6/3_O_1/1_(OH)_1/1_]^−^ layers may be the reason for the systematic threefold twinning of (NH_4_)[Ni_3_(HAsO_4_)(AsO_4_)(OH)_2_] crystals. Significant overlaps of the reflections of the respective twin domains complicated the structure solution and refinement.

## Abstract 2

The crystal structure of the title compound consists of ^2^_∞_[Ni_3_As_2_(OH)_6/3_O_18/3_O_1/1_(OH)_1/1_] layers extending parallel to (001) and exhibits disorder of the (O/OH) units of the (hydrogen)arsenate anion; the ammonium counter-cations are sandwiched between adjacent layers.

## 1. Chemical context

The natural occurrence of numerous arsenates creates a mineralogical spotlight and hence the need for a crystal-chemical classification of the respective minerals (Drahota & Filippi, 2009 ▸; Majzlan *et al.*, 2014 ▸). However, arsenate(V) minerals or synthetic compounds have been investigated not only because of their rich structural chemistry but also for their technical relevance, for example in terms of non-linear optical properties (Dhouib *et al.*, 2014 ▸, 2017 ▸) or protonic conductivity (Chouchene *et al.*, 2017*a* ▸,*b* ▸).

From the viewpoint of crystal engineering, the tetra­hedral [AsO_4_]^3–^ unit is an inter­esting, non-centrosymmetric building block that can be incorporated into transition-metal oxidotellurate(IV) frameworks. As shown for other tetra­hedral oxido-anions such as sulfate [SO_4_]^2–^ (Weil & Shirkhanlou, 2017*a* ▸,*b* ▸,*c* ▸), selenate [SeO_4_]^2–^ (Weil & Shirkhanlou, 2017*a* ▸,*b* ▸,*c* ▸) or phosphate [PO_4_]^3–^ (Zimmermann *et al.*, 2011 ▸; Eder & Weil, 2020 ▸), similar attempts were made for arsenate [AsO_4_]^3–^ anions. In this regard, the syntheses of transition-metal oxidotellurate(IV) arsenate(V) phases have previously been reported and accomplished by a chemical transport reaction for Cu_5_(TeO_3_)_2_(AsO_4_)_2_ (Missen *et al.*, 2020 ▸) and by the hydro­thermal method for Zn_2_(HTeO_3_)(AsO_4_) (Eder & Weil, 2021 ▸).

During experiments targeted at the incorporation of arsenate(III) or -(V) anions into transition-metal oxidotellurates(IV), the title compound was obtained serendipitously under hydro­thermal conditions. In the present paper, we report on the synthesis and crystal structure analysis of (NH_4_)[Ni_3_(HAsO_4_)(AsO_4_)(OH)_2_], (**I**), and its comparison with related crystal structures.

## 2. Structural commentary

The asymmetric unit of (**I**) comprises two Ni, one As, four O, one N and two H atoms; the atoms belonging to the ammonium cation could not be localized. Except for O3, which is situated on a general position (multiplicity 8, Wyckoff letter *j*), all the other atoms are at special positions of space group *C*2/*m*: Ni1 (2*a*) and N1 (2*c*) exhibit site symmetry 2/*m*, Ni2 (4*e*) site symmetry 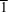 , and the remaining atoms site symmetry *m* (4*i*).

**Figure 1:**
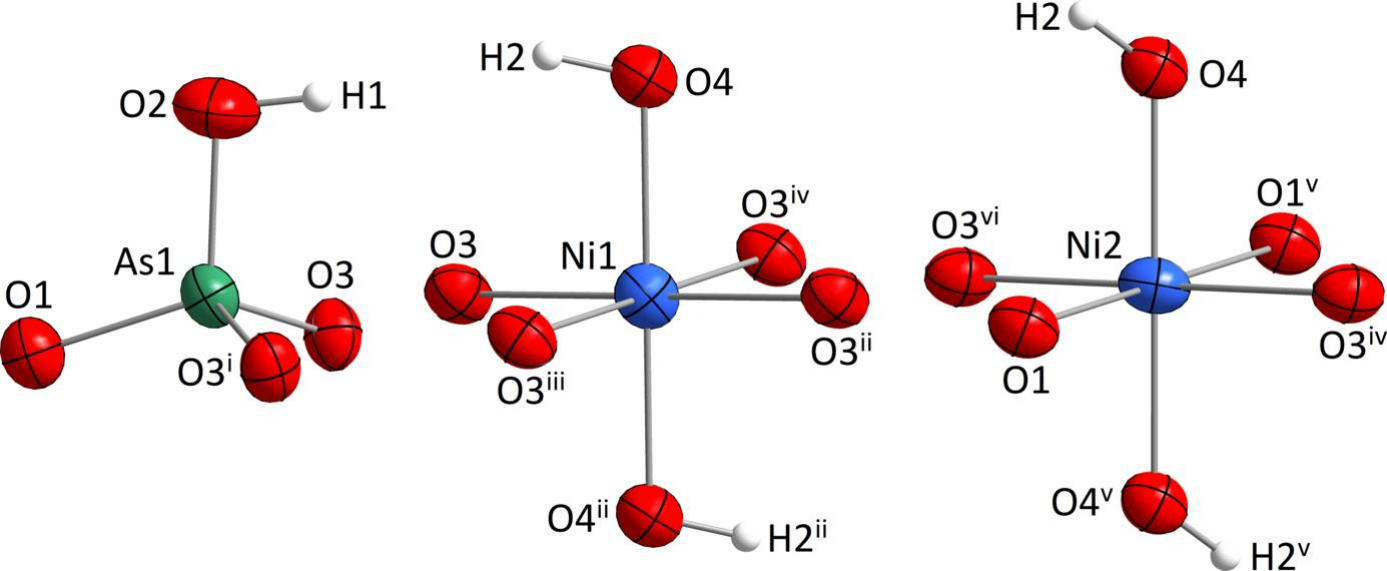
Atomic environments of the Ni^II^ and As^V^ atoms in the crystal structure of (NH_4_)[Ni_3_(HAsO_4_)(AsO_4_)(OH)_2_]. Displacement ellipsoids are drawn at the 90% probability level. Symmetry codes refer to Table 1 ▸.

**Figure 2:**
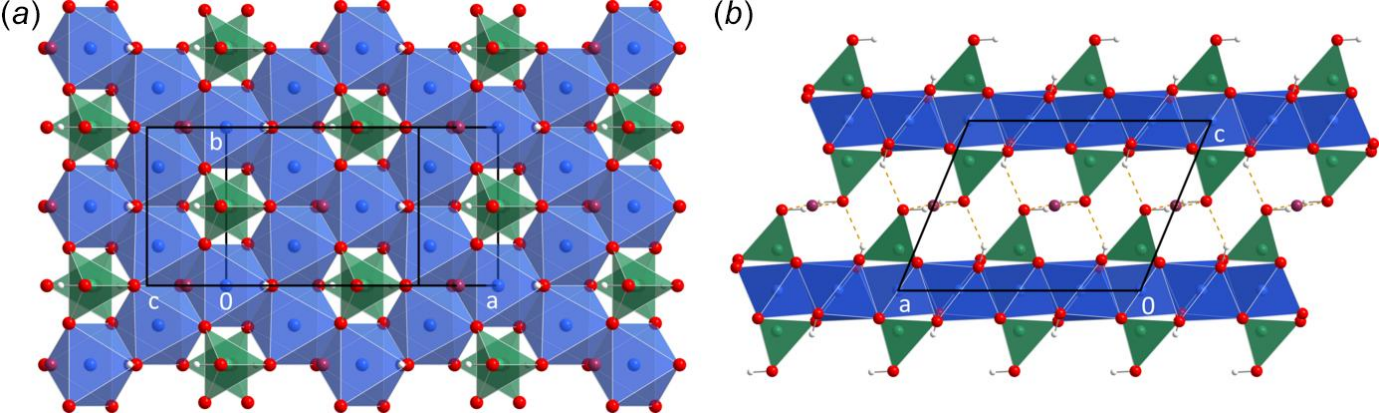
The crystal structure of (NH_4_)[Ni_3_(HAsO_4_)(AsO_4_)(OH)_2_] projected on the (001) plane (*a*) and viewed along [0 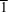 0] (*b*). Ni^II^ atoms are drawn as blue, As^V^ atoms as green, O atoms as red, N atoms as purple and H atoms as white spheres with arbitrary radius. Hydrogen bonds are drawn as orange dashed lines.

Both Ni^II^ atoms are surrounded by six oxygen atoms, two of them (O4 and its symmetry-related counterpart) being parts of hydroxide groups. The [NiO_4_(OH)_2_] units have a distorted octa­hedral shape with the hydroxide groups being in *trans* positions (Fig. 1 ▸). The OH groups have the shortest Ni—O bond lengths in both coordination polyhedra. The [NiO_4_(OH)_2_] octa­hedra are connected to four neighboring units, all by sharing edges to form ^2^_∞_[Ni_3_(OH)_6/3_O_12/2_]^8–^ layers extending parallel to (001) (Fig. 2 ▸). The O atoms and OH groups of these layers form a hexa­gonal close packed (hcp) like arrangement where 3/4 of the voids are filled with Ni^II^ atoms and 1/4 of the voids, corresponding to the Wyckoff 2*a* site, being vacant. If this void was also occupied by an Ni^II^ atom, the resulting layer resembles that present in the simple *C*6 CdI_2_ structure [also referred to as the brucite [Mg(OH)_2_] structure; Wells, 1975 ▸]. In the title compound, the As atoms are located above and below each void in the ^2^_∞_[Ni_3_(OH)_6/3_O_12/2_]^8–^ layer, sharing three oxygen atoms with the layer on either side. The bond-valence sums (BVS; Brown, 2002 ▸) of the nickel atoms were determined to be 2.06 (Ni1) and 1.99 (Ni2) valence units (v.u.) based on the parameters of Brese & O’Keeffe (1991 ▸), in good agreement with the expected value of 2.00.

The As^V^ atom in (**I**) is tetra­hedrally coordinated by oxygen atoms, spanning a range from 1.679 (10) to 1.701 (6) Å. Contrary to expectations (Schwendtner & Kolitsch, 2019 ▸), the longest As—O bond is not associated with the OH functionality (O2), which instead shows the shortest of all As—O bonds and is located at the apex of the tetra­hedron pointing away from the hexa­gonal layer. Apart from the bonded H atom, O2 solely belongs to the arsenate group and is not shared with other building blocks. The average As—O bond length is 1.694 (10) Å for the resulting [AsO_3.5_(OH)_0.5_] unit (Table 1 ▸), which is comparable to the mean As—O bond length of 1.687 (26) Å determined by Gagné & Hawthorne (2018 ▸). The BVS of the As^V^ atoms amounts to 4.88 v.u. (Brese & O’Keeffe, 1991 ▸). The H1 atom shows a short (< 0.8 Å) distance towards its own symmetry-equivalent position connected by the 2_010_ axis, and was therefore refined with a site occupation factor of 1/2. This implies that the O2 sites are equally occupied by formal O^2–^ species and the O atom of a hydroxide group. Therefore, half of the As1 atoms form [AsO_4_]^3–^ anions while the other half is present as [HAsO_4_]^2–^.

The crystal structure of (**I**) is characterized by pseudo-hexa­gonal ^2^_∞_[Ni_3_As_2_O_18/3_(OH)_6/3_O_1/1_(OH)_1/1_]^−^ layers stacked along [001]. The arsenate groups point away from the layers and form a strong asymmetric hydrogen bond between their terminal (O,OH) (O2) sites towards an [AsO_3.5_(OH)_0.5_] group of the adjacent layer [O2⋯O2 = 2.588 (18) Å]. The O4 hydroxide groups form weaker hydrogen bonds to the O2 position as well [O4⋯O2 = 2.848 (12) Å]. The remaining inter­space is occupied by the [NH_4_]^+^ cation (Fig. 2 ▸).

For the ammonium cation associated with the N1 site, no hydrogen atoms could be located. The closest oxygen atoms for hydrogen-bonding inter­actions are situated at distances of 2.930 (7) Å (4×), 3.008 (9) Å (2×) and 3.229 (4) Å (4×), respectively, which would correspond to hydrogen bonds of medium to weak strength. The site symmetry (2/*m*) of the N1 atom and the high number of possible acceptor sites for hydrogen-bonding make it most likely that the tetra­hedral [NH_4_]^+^ cation is orientationally disordered, which complicates the localization of its hydrogen atoms.

Compound (**I**) crystallizes isotypically with K[Cu_3_(HAsO_4_)(AsO_4_)(OH)_2_] (Effenberger, 1989 ▸). The relationships of the latter phase with natrochalcite, NaCu_2_(H_3_O)_2_(SO_4_)_2_ (Chevrier *et al.*, 1993 ▸) and dolerophanite, Cu_2_OSO_4_ (Effenberger, 1985 ▸), were analyzed by Effenberger (1989 ▸). Furthermore, the crystal structure of bayldonite, (Cu,Zn)_3_Pb(AsO_4_)_2_(OH)_2_ [*C*2/*c*, *a* = 10.147 (2) Å, *b* = 5.892 (1) Å, *c* = 14.081 (2) Å, *β* = 106.05 (1)°, *V* = 809.0 (2) Å^3^; Ghose & Wan, 1979 ▸], has similar metrics concerning *a* and *b* but consists of two layers per unit cell instead of one in (NH_4_)[Ni_3_(HAsO_4_)(AsO_4_)(OH)_2_]. The presence of Pb^II^ in bayldonite instead of [NH_4_]^+^ cations between the layers in the title compound results in As^V^ being exclusively present as unprotonated [AsO_4_]^3–^ anions in the structure of the mineral. Hence, hydrogen bonds are not formed between the terminal ‘O2-type’ atoms of arsenate anions belonging to adjacent layers, and the distance between neighboring ‘O2-type’ atoms is increased to 3.455 (14) Å in (Cu,Zn)_3_Pb(AsO_4_)_2_(OH)_2_ from 2.588 (18) Å in (**I**).

**Figure 3:**
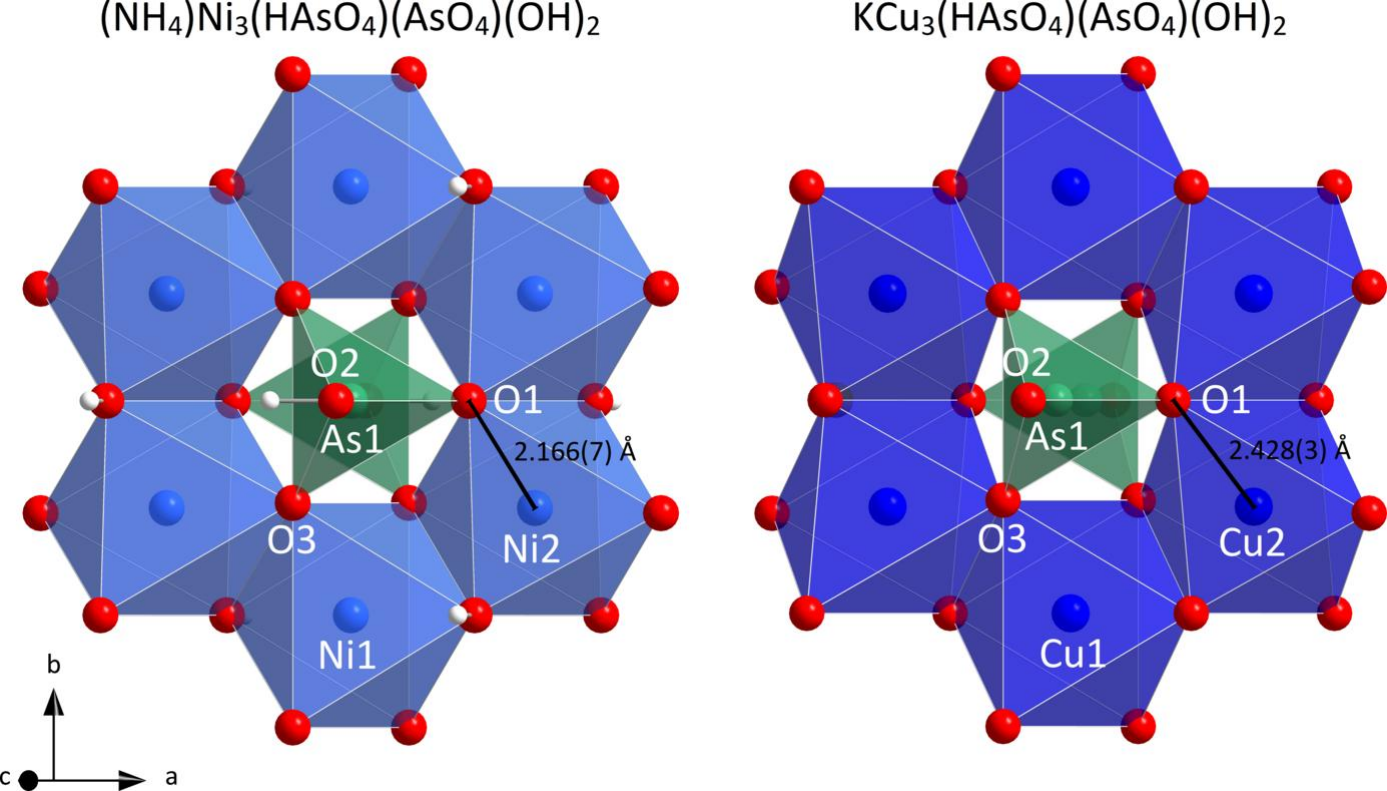
[AsO_3.5_(OH)_0.5_] and surrounding [*M*O_4_(OH)_2_] units in (NH_4_)[Ni_3_(HAsO_4_)(AsO_4_)(OH)_2_] and K[Cu_3_(HAsO_4_)(AsO_4_)(OH)_2_] projected on the (001) plane. Color codes and atomic radii refer to Fig. 2 ▸.

The crystal structure of (**I**) was qu­anti­tatively compared with K[Cu_3_(HAsO_4_)(AsO_4_)(OH)_2_] using the *compstru* software (de la Flor *et al.*, 2016 ▸) available at the Bilbao crystallographic server (Aroyo *et al.*, 2006 ▸). The degree of lattice distortion, *S*, is 0.0247, the arithmetic mean of the distances between paired atoms, *d_av_*, is 0.0926 Å, and the measure of similarity, Δ, is 0.028. For the *M*1, *M*2 and (N/K)1 sites situated on special positions, the distance between paired atoms is 0. For the other sites, values of 0.2890 Å (O1; the highest value), 0.1540 Å (O2), 0.0390 Å (O3), 0.1023 Å (O4) and 0.1178 Å (As1) were computed. Hydrogen atoms were not localized in the reference structure. The main differences between the two structures (Table 1 ▸) pertain to the higher distortion of the [CuO_6_] polyhedra in K[Cu_3_(HAsO_4_)(AsO_4_)(OH)_2_] compared to the [NiO_6_] units, which can be attributed to the strong influence of Jahn–Teller effects for Cu^II^ (Lufaso & Woodward, 2004 ▸). In particular, the Cu2 site exhibits a notable axial distortion, resulting in increased Cu2—O1 distances of 2.428 (3) Å compared to 1.934–2.000 (2) Å for the other four Cu—O bonds. These differences can also be observed in the distance distortion *ζ* (Buron-Le Cointe *et al.*, 2012 ▸) of the *M* atoms, which is 0.373 (Ni1) and 0.464 Å (Ni2) in (**I**), but is 0.765 (Cu1) and 1.229 Å (Cu2) in K[Cu_3_(HAsO_4_)(AsO_4_)(OH)_2_]. The increased Cu2—O1 distances of 2.428 (3) Å lead to a displacement of the O1 atom above the layer plane (centered at *z* = 0). This is visible from the *z* coordinate of the O1 site, which is 0.1813 (13) [compared to 0.1642 (9) for O3] in (**I**), but 0.2210 (3) [compared to 0.1672 (2) for O3] in K[Cu_3_(HAsO_4_)(AsO_4_)(OH)_2_]. This deviation leads to a stronger tilting of the [AsO_3.5_(OH)_0.5_] tetra­hedra, which results in a displacement of the As^V^ atoms from the center of the hexa­gon formed by the six surrounding *M* atoms (Fig. 3 ▸). This larger distortion with respect to the pseudo-hexa­gonal layers present in K[Cu_3_(HAsO_4_)(AsO_4_)(OH)_2_] is the supposed reason why no problems with respect to twinning features (see section 4.) were reported for the Cu-containing salt.

## 3. Synthesis and crystallization

The solid starting materials, NiO (0.1490 g; 1.99 mmol), TeO_2_ (0.1596 g; 1.00 mmol) and As_2_O_3_ (0.1974 g; 1.00 mmol), were manually mixed in a small Teflon container with an inner volume of *ca* 4 ml. Then, 0.49 g NH_3_ (aq), 25%_wt_ (7.2 mmol) and subsequently demineralized water were added to obtain a final filling degree of *ca* 3/4. The mixture was manually stirred before the container was closed with a Teflon lid. The sealed container was heated inside a steel autoclave under autogenous pressure at 483 K for one week. After cooling down to room temperature within 3 h, a grayish green solid had formed, which was filtered off and dried overnight. The reaction product was identified by powder X-ray diffraction as a mixture of elemental tellurium (≃ 50%_wt_; Bradley, 1924 ▸), responsible for the gray color, (NH_4_)[Ni_3_(HAsO_4_)(AsO_4_)(OH)_2_] (≃ 45%_wt_) and small amounts of (NH_4_)H_2_AsO_4_ (≃ 5%_wt_; Delain, 1958 ▸). This indicated that a redox reaction between the As^III^ and Te^IV^ starting materials had occurred, yielding As^V^ and elemental tellurium:

2 AsO_3_^3–^(aq) + TeO_3_^2–^(aq) + H_2_O → 2 AsO_4_^3–^(aq) + Te(s) + 2 OH^−^(aq)

In a subsequent re-synthesis, the title compound was obtained with higher yields when Ni(NO_3_)_2_(H_2_O)_6_ (0.3546 g; 1.08 mmol), As_2_O_5_(H_2_O)_*x*_ (*x* = 2–3; 0.1272 g; ≃0.46 mmol) and 0.76 g NH_3_ (aq), 25%_wt_ (10.5 mmol) were reacted under the same hydro­thermal conditions. However, the obtained material still was not single-phase; the remaining reflections could not be assigned to any literature phase, indicating another unknown phase or even phases. Crystals of (**I**) have the form of light-green blocks with sharp edges.

## 4. Refinement

Crystal data, data collection and structure refinement details are summarized in Table 2 ▸.

**Figure 4:**
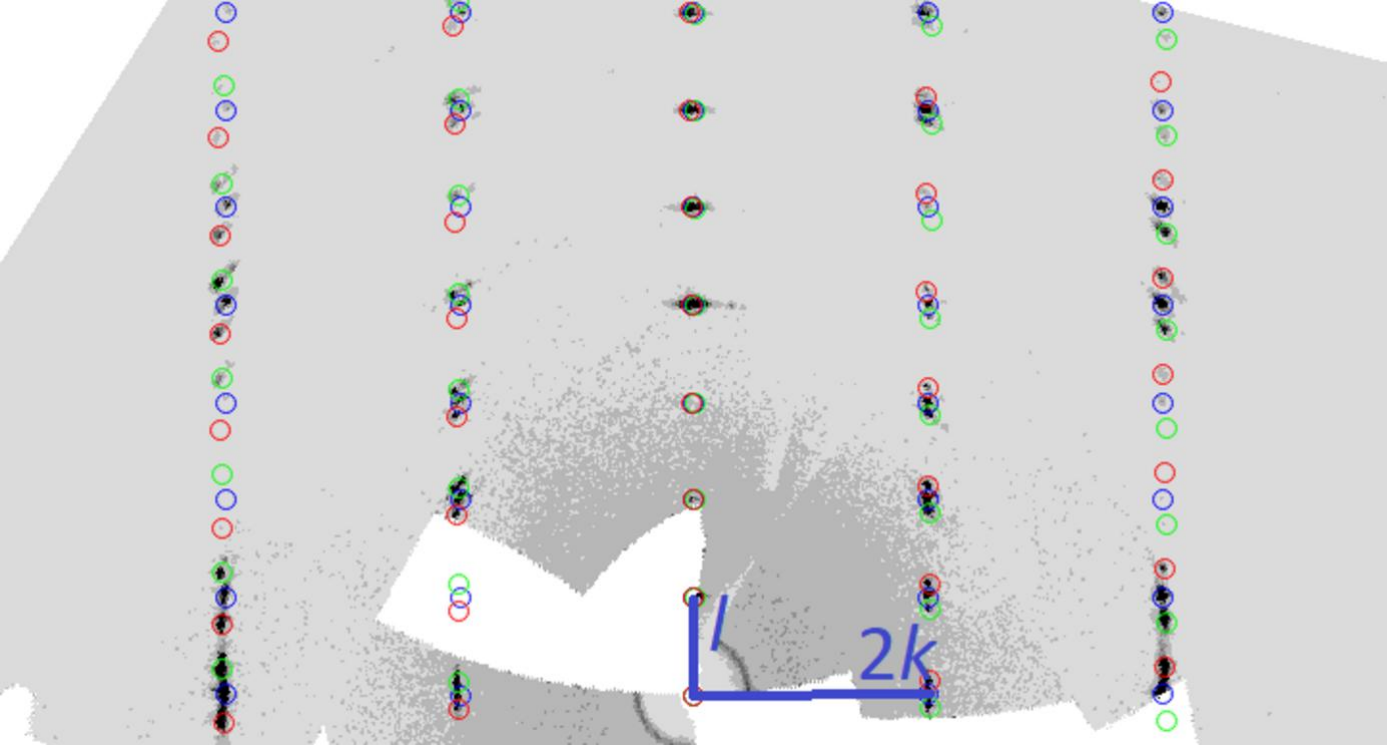
Reconstructed reciprocal 0*kl* plane of (NH_4_)[Ni_3_(HAsO_4_)(AsO_4_)(OH)_2_]. The reflections of the three twin domains are marked in blue, red and green.

**Figure 5:**
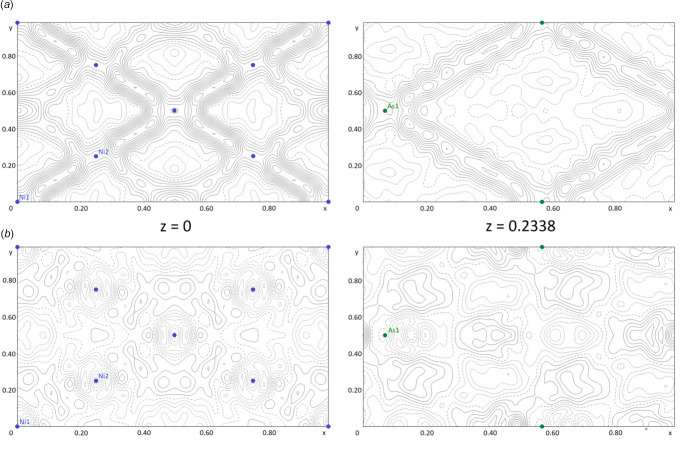
Difference contour plots of selected (001) planes in the crystal structure of (NH_4_)[Ni_3_(HAsO_4_)(AsO_4_)(OH)_2_] based (*a*) on the one-domain integration and (*b*) on the three-domain integration. Contour lines are drawn at inter­vals of 0.333 e^−^·Å^−3^ (cut-offs at −2 and 4 e^−^ Å^−3^). Plots were created with *JANA2020* (Petříček *et al.*, 2014 ▸).

All investigated crystals were systematically twinned with three domains present that are related by a 120° rotation around the c* axis (Fig. 4 ▸). The ratio of *a* and *b* (1.721) in the *C*-centered unit-cell is very close to 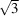 and underlines the relation to pseudo-hexa­gonality. The reflections of the corresponding domains were rather diffuse for many crystals, which resulted in the necessity of testing many crystals until one suitable for the final diffraction experiments was found. Moreover, since a significant overlap of neighboring reflections of different domains occurred frequently, the final measurement was performed with an increased sample-to-detector distance of 100 mm. Integration was attempted based either on only the most intense domain or all three domains simultaneously. The intensity data of the one-domain integration led to lower reliability factors in the resulting refinement compared to the three-domain approach (*R*_1_ = 0.050 *versus* 0.068). However, due to the overlap of reflections, disregarding the other two domains during integration led to artifacts in the resulting refinement. These features were indicated by significant difference electronic-density peaks corresponding to the positions of the heavy atoms As and Ni in the other twin domains, which resulted in a cross-shaped pattern in the difference-Fourier plots (Fig. 5 ▸ *a*). The corres­ponding three-domain integration shows significantly lower difference electron densities (Fig. 5 ▸ *b*). Despite the higher resulting reliability factors, the data resulting from the three-domain integration was chosen for the final structure refinement. The ratios of the three twin domains refined to values of 0.653 (4):0.264 (4):0.093 (2). The CIF resulting from the one-domain integration can be found in the electronic supplementary information (ESI) for this article.

The H atoms attached to O2 and O4 were located in difference-Fourier maps. Their O—H distances were restrained to a value of 0.89 Å using the DFIX command in *SHELXL* (Sheldrick, 2015*b* ▸) and with *U*_iso_(H) = 1.5*U*_eq_(O). Atom labels and coordinates were assigned in accordance with isotypic K[Cu_3_(HAsO_4_)(AsO_4_)(OH)_2_] (Effenberger, 1989 ▸).

The only atom breaking the *C*2/*m* symmetry is atom H1 (under assumption of full occupancy). Because adjacent H1 sites are symmetrically connected by the 2_010_ axis, it was attempted to resolve the disorder of H1 by a symmetry reduction to *Cm* with inclusion of the removed symmetry operation as the twin law. In the lower-symmetric space group, the As1 and O2 sites are both split into two positions. Extensive modeling attempts in space group *Cm* based on both one-domain and three-domain integrations were performed, but the disorder of the H1 atom could not be resolved on basis of the two data sets. In general, the *Cm* models were of inferior quality due to over-parametrization and strong correlations between atom pairs, resulting in significantly larger standard uncertainties of atomic coordinates, inter­atomic distances and negative displacement parameters for some atoms. Hence, *C*2/*m* was chosen as the space group of the final model, assuming an equal distribution of O and OH at the O2 site.

Single crystals of (NH_4_)[Ni_3_(HAsO_4_)(AsO_4_)(OH)_2_] were investigated at both room temperature and 100 K, but no ordering of the hydrogen atoms was observed at the lower temperature.

In the final model (three-domain integration), the highest remaining positive and negative electron density peaks are located at the Ni1 site and 1.77 Å from O1, respectively.

## Supplementary Material

Crystal structure: contains datablock(s) I, global. DOI: 10.1107/S2056989024003487/hb8094sup1.cif

Structure factors: contains datablock(s) I. DOI: 10.1107/S2056989024003487/hb8094Isup2.hkl

CIF for the one-domain integration. DOI: 10.1107/S2056989024003487/hb8094sup3.txt

CCDC reference: 2349363

Additional supporting information:  crystallographic information; 3D view; checkCIF report

## Figures and Tables

**Table 1:** Comparison of structure data and inter­atomic distances in the crystal structures of (NH_4_)[Ni_3_(HAsO_4_)(AsO_4_)(OH)_2_] and isotypic K[Cu_3_(HAsO_4_)(AsO_4_)(OH)_2_] (Effenberger, 1989 ▸)

	(NH_4_)[Ni_3_(HAsO_4_)(AsO_4_)(OH)_2_]	[KCu_3_(HAsO_4_)(AsO_4_)(OH)_2_]
*a* (Å)	10.178 (2)	10.292 (5)
*b* (Å)	5.9156 (11)	5.983 (3)
*c* (Å)	7.7158 (14)	7.877 (4)
*β* (°)	112.658 (14)	117.86 (2)
*V* (Å^3^)	428.71 (15)	428.82
*d* (Å)		
As1—O2	1.679 (10)	1.704 (3)
As1—O1	1.693 (9)	1.661 (4)
As1—O3	1.701 (6)	1.704 (2)
As1—O3^i^	1.701 (6)	1.704 (2)
*M*1—O4	1.965 (8)	1.899 (2)
*M*1—O4^ii^	1.965 (8)	1.899 (2)
*M*1—O3	2.100 (6)	2.186 (2)
*M*1—O3^ii^	2.100 (6)	2.186 (2)
*M*1—O3^iii^	2.100 (6)	2.186 (2)
*M*1—O3^iv^	2.100 (6)	2.186 (2)
*M*2—O4	1.954 (6)	1.934 (2)
*M*2—O4^v^	1.954 (6)	1.934 (2)
*M*2—O3^iv^	2.099 (6)	2.000 (2)
*M*2—O3^vi^	2.099 (6)	2.000 (2)
*M*2—O1	2.166 (7)	2.428 (3)
*M*2—O1^v^	2.166 (7)	2.428 (3)
O2—H1	0.9 (3)	–
O2⋯O2^vii^	2.588 (18)	2.491 (5)
O4—H2	0.90 (7)	–
O4⋯O2^viii^	2.848 (12)	2.692 (3)

Symmetry codes: (i) *x*, 1 − *y*, *z*; (ii) −*x*, −*y*, *z*; (iii) *x*, −*y*, *z*; (iv) −*x*, *y*, −*z*; (v) 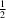 − *x*, 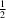 − *y*, −*z*; (vi) 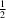 + *x*, 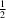 − *y*, *z*; (vii) −*x*, *y*, 1 − *z*; (viii) 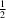 − *x*, − 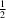 + *y*, 1 − *z*.

**Table 2:** Experimental details

Crystal data	
Chemical formula	(NH_4_)[Ni_3_(HAsO_4_)(AsO_4_)(OH)_2_]
*M*_r_	507.04
Crystal system, space group	Monoclinic, *C*2/*m*
Temperature (K)	296
*a*, *b*, *c* (Å)	10.178 (2), 5.9156 (11), 7.7158 (14)
β (°)	112.658 (14)
*V* (Å^3^)	428.71 (15)
*Z*	2
Radiation type	Mo *K*α
μ (mm^−1^)	14.23
Crystal size (mm)	0.05 × 0.04 × 0.03

Data collection	
Diffractometer	Stoe Stadivari CCD
Absorption correction	Multi-scan (*LANA*; Koziskova *et al.*, 2016 ▸)
*T*_min_, *T*_max_	0.281, 0.345
No. of measured, independent and observed [*I* > 2σ(*I*)] reflections	3235, 3235, 2214
(sin θ/λ)_max_ (Å^−1^)	0.814

Refinement	
*R*[*F* ^2^ > 2σ(*F* ^2^)], *wR*(*F* ^2^), *S*	0.068, 0.229, 1.04
No. of reflections	3235
No. of parameters	54
No. of restraints	2
H-atom treatment	Only H-atom coordinates refined
Δρ_max_, Δρ_min_ (e Å^−3^)	2.95, −2.30

Computer programs: *X-AREA* (Stoe, 2021 ▸), *SHELXT* (Sheldrick, 2015*a* ▸), *SHELXL* (Sheldrick, 2015*b* ▸), *DIAMOND* (Brandenburg, 2016 ▸) and *publCIF* (Westrip, 2010 ▸).

## supplementary crystallographic information

### Crystal data

**Table:**

(NH_4_)[Ni_3_(HAsO_4_)(AsO_4_)(OH)_2_]	*F*(000) = 488
*M**_r_* = 507.04	*D*_x_ = 3.928 Mg m^−^^3^
Monoclinic, *C*2/*m*	Mo *K*α radiation, λ = 0.71073 Å
*a* = 10.178 (2) Å	Cell parameters from 7257 reflections
*b* = 5.9156 (11) Å	θ = 4.1–35.9°
*c* = 7.7158 (14) Å	µ = 14.23 mm^−^^1^
β = 112.658 (14)°	*T* = 296 K
*V* = 428.71 (15) Å^3^	Block, light green
*Z* = 2	0.05 × 0.04 × 0.03 mm

### Data collection

**Table:**

Stoe Stadivari CCD diffractometer	3235 independent reflections
Radiation source: Axo_Mo	2214 reflections with *I* > 2σ(*I*)
rotation method, ω scans	θ_max_ = 35.3°, θ_min_ = 2.9°
Absorption correction: multi-scan (*LANA*; Koziskova *et al.*, 2016)	*h* = −16→16
*T*_min_ = 0.281, *T*_max_ = 0.345	*k* = −9→9
3235 measured reflections	*l* = −12→12

### Refinement

**Table:**

Refinement on *F*^2^	2 restraints
Least-squares matrix: full	Hydrogen site location: difference Fourier map
*R*[*F*^2^ > 2σ(*F*^2^)] = 0.068	Only H-atom coordinates refined
*wR*(*F*^2^) = 0.229	*w* = 1/[σ^2^(*F*_o_^2^) + (0.1507*P*)^2^] where *P* = (*F*_o_^2^ + 2*F*_c_^2^)/3
*S* = 1.04	(Δ/σ)_max_ < 0.001
3235 reflections	Δρ_max_ = 2.95 e Å^−^^3^
54 parameters	Δρ_min_ = −2.29 e Å^−^^3^

### Special details

**Table:**

Geometry. All esds (except the esd in the dihedral angle between two l.s. planes) are estimated using the full covariance matrix. The cell esds are taken into account individually in the estimation of esds in distances, angles and torsion angles; correlations between esds in cell parameters are only used when they are defined by crystal symmetry. An approximate (isotropic) treatment of cell esds is used for estimating esds involving l.s. planes.
Refinement. Refined as a 3-component twin.

### Fractional atomic coordinates and isotropic or equivalent isotropic displacement parameters (Å^2^)

**Table:**

	*x*	*y*	*z*	*U*_iso_*/*U*_eq_	Occ. (<1)
Ni1	0.000000	0.000000	0.000000	0.0123 (4)	
Ni2	0.250000	0.250000	0.000000	0.0125 (4)	
As1	0.06863 (12)	0.500000	0.23948 (15)	0.0106 (3)	
O1	0.2130 (9)	0.500000	0.1813 (13)	0.0131 (14)	
O2	0.1181 (11)	0.500000	0.4737 (14)	0.0211 (19)	
O3	−0.0309 (6)	0.2619 (10)	0.1642 (9)	0.0130 (10)	
O4	0.2077 (9)	0.000000	0.1357 (12)	0.0128 (14)	
N1	0.000000	0.000000	0.500000	0.024 (3)	
H1	0.03 (2)	0.500000	0.48 (7)	0.036*	0.5
H2	0.22 (2)	0.000000	0.257 (9)	0.036*	

### Atomic displacement parameters (Å^2^)

**Table:**

	*U*^11^	*U*^22^	*U*^33^	*U*^12^	*U*^13^	*U*^23^
Ni1	0.0132 (9)	0.0101 (8)	0.0143 (10)	0.000	0.0063 (7)	0.000
Ni2	0.0136 (6)	0.0102 (6)	0.0153 (7)	0.0001 (4)	0.0072 (5)	0.0014 (4)
As1	0.0118 (5)	0.0088 (5)	0.0127 (5)	0.000	0.0062 (4)	0.000
O1	0.014 (3)	0.012 (3)	0.016 (4)	0.000	0.007 (3)	0.000
O2	0.024 (4)	0.028 (5)	0.012 (4)	0.000	0.007 (3)	0.000
O3	0.012 (2)	0.011 (2)	0.016 (3)	−0.0018 (18)	0.0063 (19)	−0.0019 (18)
O4	0.012 (3)	0.012 (3)	0.015 (4)	0.000	0.006 (3)	0.000
N1	0.037 (9)	0.016 (6)	0.018 (7)	0.000	0.011 (6)	0.000

### Geometric parameters (Å, º)

**Table:**

Ni1—O4	1.965 (8)	Ni2—O3^v^	2.099 (6)
Ni1—O4^i^	1.965 (8)	Ni2—O3^iii^	2.099 (6)
Ni1—O3^i^	2.100 (6)	Ni2—O1	2.166 (7)
Ni1—O3^ii^	2.100 (6)	Ni2—O1^iv^	2.166 (7)
Ni1—O3^iii^	2.100 (6)	As1—O2	1.679 (10)
Ni1—O3	2.100 (6)	As1—O1	1.693 (9)
Ni2—O4^iv^	1.954 (6)	As1—O3^vi^	1.701 (6)
Ni2—O4	1.954 (6)	As1—O3	1.701 (6)
			
O4—Ni1—O4^i^	180.0	O3^v^—Ni2—O1	92.4 (3)
O4—Ni1—O3^i^	86.6 (2)	O3^iii^—Ni2—O1	87.6 (3)
O4^i^—Ni1—O3^i^	93.4 (2)	O4^iv^—Ni2—O1^iv^	92.4 (3)
O4—Ni1—O3^ii^	93.4 (2)	O4—Ni2—O1^iv^	87.6 (3)
O4^i^—Ni1—O3^ii^	86.6 (2)	O3^v^—Ni2—O1^iv^	87.6 (3)
O3^i^—Ni1—O3^ii^	84.9 (3)	O3^iii^—Ni2—O1^iv^	92.4 (3)
O4—Ni1—O3^iii^	86.6 (2)	O1—Ni2—O1^iv^	180.0 (4)
O4^i^—Ni1—O3^iii^	93.4 (2)	O2—As1—O1	110.7 (5)
O3^i^—Ni1—O3^iii^	95.1 (3)	O2—As1—O3^vi^	105.1 (3)
O3^ii^—Ni1—O3^iii^	180.0 (3)	O1—As1—O3^vi^	111.9 (3)
O4—Ni1—O3	93.4 (2)	O2—As1—O3	105.1 (3)
O4^i^—Ni1—O3	86.6 (2)	O1—As1—O3	111.9 (3)
O3^i^—Ni1—O3	180.0	O3^vi^—As1—O3	111.8 (4)
O3^ii^—Ni1—O3	95.1 (3)	As1—O1—Ni2	123.8 (3)
O3^iii^—Ni1—O3	84.9 (3)	As1—O1—Ni2^vii^	123.8 (3)
O4^iv^—Ni2—O4	180.0	Ni2—O1—Ni2^vii^	86.1 (3)
O4^iv^—Ni2—O3^v^	87.0 (3)	As1—O3—Ni2^iii^	126.1 (3)
O4—Ni2—O3^v^	93.1 (3)	As1—O3—Ni1	127.5 (3)
O4^iv^—Ni2—O3^iii^	93.0 (3)	Ni2^iii^—O3—Ni1	89.0 (2)
O4—Ni2—O3^iii^	86.9 (3)	Ni2^viii^—O4—Ni2	98.4 (4)
O3^v^—Ni2—O3^iii^	180.0	Ni2^viii^—O4—Ni1	97.4 (3)
O4^iv^—Ni2—O1	87.6 (3)	Ni2—O4—Ni1	97.4 (3)
O4—Ni2—O1	92.4 (3)		

Symmetry codes: (i) −*x*, −*y*, −*z*; (ii) *x*, −*y*, *z*; (iii) −*x*, *y*, −*z*; (iv) −*x*+1/2, −*y*+1/2, −*z*; (v) *x*+1/2, −*y*+1/2, *z*; (vi) *x*, −*y*+1, *z*; (vii) −*x*+1/2, *y*+1/2, −*z*; (viii) −*x*+1/2, *y*−1/2, −*z*.
